# Herholdt's Observations on Respiration

**Published:** 1801-11

**Authors:** 


### *Herholdt's Observations on Respiration.*

MR. HERHOLDT, a very able ſurgeon at Copenhagen, has lately publiſhed an intereſting work on the Surgical Treatment of deep Wounds of the Breaſt, from the phyſiological part of which we communicate the following obſervations.

It has been aſſerted by ſome Naturaliſts, that the fetus, during its ſtay in the uterus, inſpires the liquor amnii, by which it is ſurrounded; an opinion which ſeems to be founded on the following phenomena. — 1. The fetus has been obſerved to move the breaſt and belly in the liquor amnii, like a perſon breathing. 2. The chicken in the egg ſeems to draw in the white or albumen, and even to chirp, a few days before it breaks the ſhell.

In oppoſition to this opinion, Mr. H. has made ſeveral experiments and obſervations, by which it appears ill grounded. He put ſome puppies, before they had begun breathing, into a veſſel full of milk, in which he ſaw them continue the abovementioned motions of the breaſt till they died; but on opening them, he did not find them to have drawn in a ſingle drop of the milk It is ſtill farther improbable that the cauſe why the chicken chirps in the egg ſhould be aſcribed to the undulated motions of the albumen, as has been pretended; for as long as its beak is ſurrounded with albumen, it has not the power of emitting any ſound. In the laſt days of incubation, however, when the chicken is increaſing, a conſiderable air bladder forms itſelf at the broad end of the egg, which the chicken generally perforates with its beak ſome time before it breaks the ſhell; from which circumſtance it appears, that the air contained in that that bladder has penetrated into the lungs of the animal before the chirping found is perceived. In order to afcertain this, Mr. H. made the following experiment: He befmeared fome eggs, in which the chickens had been chirping, with oil, and placed them in warm milk till the animals died. On opening them, he always found that the beak had penetrated the membrane of the air bladder, and the lungs were fo porous and light as to fwim on the furface of water. Another argument againft that opinion may be taken from fome monfters in the animal creation, which, though without a head, nofe, or mouth, continued to live and to grow in the uterus of the mother. Mr. Portal found the windpipe and its ramifications in three full grown fetuffes to be filled with a thick gelatine, which had moft probably occafioned their death. Now, how could this gelatine have been accumulated in the trachea, or refted there fo long as to receive that degree of toughnefs, if the liquor amnii did pafs and repafs the lungs like the air in animals that have come into the world? Or why was it only in the trachea, and not in the very fubftance of the lungs? All which phenomena feem to prove how much the above opinion recedes from truth and experience.

The caufe of beginning refpiration is generally thought to originate in the firft impreffion of the air on the rima glottidis, though it has not yet been afcertained whether the atmofphere merely acts here as an incitement, or by yielding a particle, through means of which the diaphragm is enabled to contract itfelf; which laft circumftance Mr. H. thinks highly probable, becaufe the nerves of the rima glottidis being moft intimately connected with the diaphragm, are likely to receive oxygen, electricity, &c. from the atmofphere, and thus to excite this organ to action. This ingenious conjecture, firft intimated by Abildgaard, feems to obtain fome probability by the following obfervation. It is known from experiments, that if a ligature be applied to the windpipe of an animal whofe lungs are previoufly filled with oxygen gas, the animal endeavours to breathe with the fame effort as when the lungs had been filled with azotic gas; which phenomena moft likely originates from the rima glottidis remaining in contact with the atmofphere in both cafes. An additional argument for this conjecture may be taken from animals which are enclofed in irrefpirable gaffes, as they are only capable of expiring when infpiration ceafes entirely, and the animal dies before the pulfation is ftopped. It may be farther fuggefted, that drowned people are frequently reftored to life by proper treatment, without having blown air into their lungs; and likewife hanged people immediately die, when they are ftrangulated above the rima glottidis; whereas the organs of refpiration continue to act till the pulfe has ceafed and till the nervous fyftem is difordered, when the windpipe has been tied more downwards.

The lungs do not evolve themfelves till after the birth; before that time they will not fall together on opening the pleura, but they lie in the breaft as a folid and heavy mafs, and when put into water they fink to the bottom; and no bubble rifes on their being cut into under water. Placed under the air-pump in which the air is rarified, they do not increafe in fize; all which fhows that neither air nor any liquor is contained in the veficles of the lungs.

In this ftate they take up but little room, the cavum thoracis being at the fame time not fo great proportionably as in a breathing fubject, and the diaphragm confiderably vaulted; neither air can enter the breaft, nor the lungs be preffed out, when an opening is made into the breaft; in fhort, the capacity of the breaft in the fetus, ftands in determined proportion to the abfolute fize of the organs which are enclofed in it. But on the child coming into the world, and the liquor amnii having quited the throat, the atmofphere excites the organs of refpiration to action, the diaphragm begins to contract itfelf, the mufcles raife the ribs upwards, and the breaft is enlarged; this motion is followed by an oppofite one, by which the cavity of the breaft decreafes, and the lungs are contracted proportionably to the cavity of the cavum thoracis; during the firft period a part of the atmofphere enters into the lungs, but is expelled by the oppofite motion. The child has hardly begun to breathe, when confiderable changes take place in his lungs; the alteration in the circulation of the blood has increafed their abfolute weight, while the fpecific weight is confiderably diminifhed; they take up more room, receive a different colour, and a veficular appearance, and keep fwimming on the furface of pure water, in which when they are preffed they emit bubbles, and they grow confiderably larger in air rarified by the air-pump.

The quantity of air in the breaft increafes, when with the growth of the body the lungs become more capacious. It has however not yet been exactly determined what quantity of air might be received into the lungs; and though feveral experiments were made by naturalifts for that purpofe, no certain rule could be fixed, but the refults are in general the following: 1. The lungs taken out from the body of an adult can be faid to contain about 6 cubic inches of air. 2. On opening both facci pleuræ the atmofphere enters through the wounds into the breaft, while the lungs contract themfelves towards the backbone, and emit a quantity of air through the windpipe, pipe, which circumſtance ſeems to prove that the lungs contain a great deal more air before the opening in the breaſt was made. The whole maſs of air that is contained in the lungs of an adult after expiration, amounts moſt probably to about 110 cubic inches. 3. This quantity of air is increaſed by inſpiration, for each time of which about 40 inches are requiſite, in a quiet ſtate and in a pure air of moderate temperature; ſo that, conſequently, the lungs of a man contain about 150 cubic inches of air after a common inſpiration. 4. The greateſt quantity of air which the man is able to inſpire with a violent effort of the organs for reſpiration, may be fixed to 172 cubic inches, and the proportion betwixt the quantity after expiration, and that after inſpiration, is conſequently as 110:62.

The motion of the lungs during reſpiration, is univerſally allowed to be occaſioned by the diaphragm and the intercoſtal muſcles, which by their cotemporary action widen the breaſt, where, afterwards, the air enters into the lungs, and which may be conſidered as active organs of inſpiration. But whether inſpiration merely depends on the preſſure of the atmoſphere during the enlargement of the breaſt, or whether the lungs poſſeſs a peculiar power of imbibing air, is a matter of enquiry and diſcuſſion, upon which Mr. H. enlarges; ſhowing, that the lungs are moſt probably deprived of any active part during inſpiration, as will appear from the following remarks. It is true that frogs ſeem to extend their lungs without the help of the diaphragm, with which they are not provided, the cauſe of which phenomenon however, ariſes from the peculiar organization of its inſtruments for reſpiration, but not from a particular power of the lungs. The breathing of a frog proceeds in the following way. After having drawn in the air through the noſtrils, he cloſes them, and by raiſing the trachea, he diminiſhes the capacity of the mouth, whereby the air being compreſſed, enters through the open windpipe, where it finds the leaſt reſiſtance, into the veſicular lungs. A frog therefore inſpires by widening its mouth, and ſhutting it cloſely, which if he is prevented to do, the lungs collapſe and are not capable of being extended; thus one may kill frogs by putting a ſmall ſtick into the mouth, ſo as to prevent their cloſing it. *Mr. Hook* took the ribs, the diaphragm, and the pericardium from a living animal, upon which the lungs immediately collapſed, reſpiration ceaſed, and the animal became almoſt lifeleſs. Having cut off the windpipe, and blown air into the lungs with a pair of bellows, an artificial reſpiration was produced, and the life of the animal preſerved for more than an hour. *Mr. Herholdt* himſelf made the following experiment, which evidently proves that the lungs have no active power in inſpiration. He took a kitten a kitten the moment it was brought into the world, and having ſtopped the noſe and mouth, he brought the head under water, and made with proper care two large wounds on each ſide of the breaſt through the pleura. On taking it out of the water, it was ſtill living, and began immediately to move the breaſt, the air paſſing and repaſſing the wounds, but not the leaſt draught of air was to be perceived before the noſe. On opening the body after death, the lungs were found to be in the ſame ſtate as in a kitten not born, and not containing the leaſt air, becauſe the widening of the breaſt could not act on the lungs, on account of the wounds that were made in them. The caſe is quite different during the act of expiration; when the breaſt is filled with air, and the muſcles for inſpiration ceaſe to act, the lungs endeavour to expel the air by means of their contractility, in combination with the weight of the pectoral muſcles and thoſe of the belly, and thus to diminiſh their ſize. This contractility they keep, even for ſome time after death.

